# Is a Coded Physical Activity Diary Valid for Assessing Physical Activity Level and Energy Expenditure in Stroke Patients?

**DOI:** 10.1371/journal.pone.0098735

**Published:** 2014-06-06

**Authors:** Christel Vanroy, Yves Vanlandewijck, Patrick Cras, Hilde Feys, Steven Truijen, Marc Michielsen, Dirk Vissers

**Affiliations:** 1 Department of Rehabilitation Sciences and Physiotherapy, Faculty of Medicine and Health Sciences, University of Antwerp, Antwerp, Belgium; 2 Translational Neurosciences, Faculty of Medicine and Health Sciences, University of Antwerp, Antwerp, Belgium; 3 KU Leuven, Department of Rehabilitation Sciences, Leuven, Belgium; 4 Department of Neurology, Antwerp University Hospital, Antwerp, Belgium; 5 Department of Neurology, Born-Bunge Institute, Antwerp, Belgium; 6 Jessa Hospital, Rehabilitation campus Sint- Ursula, Herk-de-Stad, Belgium; University of Glasgow, United Kingdom

## Abstract

**Objectives:**

to determine the concurrent validity of a physical activity diary for measuring physical activity level and total energy expenditure in hospitalized stroke patients.

**Method:**

Sixteen stroke patients kept coded activity diaries and wore SenseWear Pro2 multi-sensor activity monitors during daytime hours for one day. A researcher observed the patients and completed a diary. Data from the patients' diaries were compared with observed and measured data to determine total activity (METs*minutes), activity level and total energy expenditure.

**Results:**

Spearman correlations between the patients' and researchers' diaries revealed a high correlation for total METs*minutes (r_s_ = 0.75, p<0.01) for sedentary (r_s_ = 0.74,p<0.01) and moderate activities (r_s_ = 0.71,p<0.01) and a very high correlation (r_s_ = 0.92, p<0.01) for the total energy expenditure. Comparisons between the patients' diaries and activity monitor data revealed a low correlation (r_s_ 0.29) for total METs*minutes and energy expenditure.

**Conclusion:**

Coded self-monitoring activity diaries appear feasible as a low-tech alternative to labor-intensive observational diaries for determining sedentary, moderate, and total physical activity and for quantifying energy expenditure in hospitalized stroke patients. Given the poor correlation with objective measurements of physical activity, however, further research is needed to validate its use against a gold-standard measure of physical activity intensity and energy expenditure.

## Introduction

The importance of physical activity promoting health has been well documented. Increased daily physical activity reduces cardiovascular risk for people with and without disabilities [1], [2]. Stroke patients have reduced levels of physical activity due to the nature of their impairments. Several observational studies have described decreases in the activity patterns of sub-acute and chronic stroke patients [2]–[4]. There is considerable interest in exploring valid and reliable instruments for evaluating the level of daily physical activity and in identifying physical activity patterns to guide intervention strategies.

A variety of objective methods have been used to measure daily physical activity in stroke patients, including activity monitors [5]–[7] and pedometers [8]–[10]. Activity monitors based on accelerometry, measure acceleration, as expressed in energy expenditure and/or ambulatory movement. Advantages of activity monitors include their objectivity and the fact that they do not rely on cognitive/memory skills. Activity monitoring also allows the possibility of testing a large sample, and recording continuously for long periods under free-living conditions [7]. Commonly reported major shortcomings include the loss of data due to noncompliance and the failure of activity monitors due to malfunctioning or loosening of the equipment [6], [11]–[13]. In addition, hemiparetic gait disturbances and/or arm movements causes unreliable recordings in accelerometry systems [9], [10], [14].

Pedometer are prescribed as a less expensive and simple alternative for taking objective measurements of physical activity in stroke patients [8], [15]. Pedometers tend to undercount steps during slower gaits when the device is placed on the hip. In hemiparetic gait, speed accelerations at the hip were often of insufficient magnitude to be registered [8]. A knee-worn pedometer has recently been recommended for detecting all walking activities in stroke patients, with the exception of high intensity walking [10]. One explanation might be that, in hemiparetic gait, the knee joint shows more vertical acceleration, thus bringing the lever arm in contact with the electrical component of the device. Pedometers detect only the number of steps and provide no information about energy expenditure or the type and intensity of activities performed by patients.

Other methods for determining physical activity in stroke patients include observation [16], physical activity questionnaires [17]–[19] and activity diaries [20], [21]. Observational methods require a researcher to observe a patient at set intervals over a specified period, in order to produce reliable data [16], [22]. This method is time consuming and costly, and it is therefore less applicable in clinical settings. Activity questionnaires and diaries have the advantage of low cost and suitability for comparison between populations. Questionnaires are the most frequently used instruments in epidemiological studies for estimating physical activity and energy expenditure [17]–[19]. Although they save time, these questionnaires rely on retrospective information and honest reporting, and they do not allow for cognitive deficit. Questionnaires with greater detail are used for assessing the duration, frequency, and intensity of activity. Because of their complexity, however, they often result in lower compliance and lower validity [23]. Although questionnaires with less detail are easier to use, they are often less accurate, and they do not assess various dimensions of physical activity [24]. In stroke research, activity diaries are most commonly used as secondary outcome measurements, given the difficulty of recording activities due to patients impairments [6]. In healthy participants; a categorized three-day physical activity diary was used as an alternative method for assessing various dimensions of physical activity. Each day was divided into 96 15-minute intervals. The participants were asked to grade their activity into nine categories (cat.1 = sleeping, cat.2 = sitting, cat.3 = standing, cat.4 = walking inside, cat.5 = walking outside, cat.6 9 = low, moderate, high, and very high intensity activity, respectively) [25]. Participants were asked to choose one dominant activity for each 15-minute period. This type of diary has been described as being time efficient, easy to learn, inexpensive, reliable, and reasonably valid [20], [26], [27]. One disadvantage of the three-day diary is that it underestimates activities of short duration, as it records only the major activity performed during each 15-minute period was recorded [20]. Researchers have noted that participants are unable to keep with the diary if periods of 5–10 minutes periods are used. Another difficulty involves the limited choice in activities. Researchers have concluded that the diary is an alternative method for evaluating individual physical patterns and that it is suitable for clinical practice in healthy participants.

For stroke patients, a tool is needed that combines the advantages of the three-day categorized-activity diary with greater detail information about the type and intensity of activities and the position in which the activities are executed, in order to provide accurate information with minimal effort, thus being useful in clinical settings. In stroke patients, activities of short duration occur rarely, if at all. The short time intervals recommended in the Bouchard study are therefore not preferable. Moreover, therapy in rehabilitation centers is often scheduled in 30-minutes blocks. Keeping the diary can help patients to become more aware of their physical activities, possibly strengthening their motivation to adopt a more active lifestyle. To this end, a simplified coded physical-activity diary was developed in which stroke patients choose the dominant activity in performed 30-minute time interval from a pre-defined list of activities, all linked to simple codes. This minimizes writing, making it possible for patients with writing problems to complete the diary. The time was adjusted to the pace of hospitalized stroke patients, who perform fewer activities within 30-minute time interval in a rehabilitation center.

To our knowledge, no study in stroke research has investigated the use of a coded self-monitoring activity diary to determine both total energy expenditure and intensity level of various activities, compared against criterion standards of observations and activity monitoring. In the present study the concurrent validity of an activity diary was evaluated in hospitalized stroke patients. We specifically compared self-monitoring diaries to observational diaries and activity-monitor outcomes.

## Methods

### Ethics Statement

The protocol was reviewed and approved by the Medical Ethics Committee of the Antwerp University Hospital, Belgium (no. B30020084906). Patients received oral and written information about the design of the study; they provided written consent and agreed to the publication of the research data.

### Participants

Stroke patients were recruited on a voluntary basis from an inpatient rehabilitation center in Belgium. Inclusion criteria were as follows: (1) a first-ever stroke as defined by the World Health Organization, (2) stroke less than six months ago, (3) ability to move independently with or without a walking aid and (4) understand and carry out simple instructions. Patients were excluded if they were not medically stable, as described by the American College of Cardiology Foundation/American Heart Association [28].

### Design

On the first day demographic and clinical data were collected, including age, gender, duration and type of stroke, height, weight and the degree of loss of function (Rivermead Motor Assessment, Gross function [29]). The patients also had an introductory session with the equipment on this day. The SWP2A was placed on the non-hemiplegic arm and patients were told not to take off the monitor until the end of the study period. They also received instructions on completing the diary. The following day, all patients were asked to complete a daytime activity diary simultaneously, in addition to wearing an SWP2A.

After receiving instructions on completing the diary, each patient entered one activity diary independently, while another diary was completed by an observer, both between 8:00 AM and 8:00 PM. This timeframe was selected because patients were considered most active between these hours in rehabilitation centers. The patients were asked to list their main activities for each half hour. A researcher observed each patient once every 20 minutes, completing the observer activity diary independently. The following day, both diaries and the activity monitors were collected. Missing data in the patients' diaries were completed based on the recollections of the interviewer, independently of the observer. To test for concurrent validity, the patients' diaries were compared against two criterion measurements, the observers' diaries and the activity-monitor data.

### Assessment

The coded activity diary was developed based on two existing activity diaries [24], [30]. The simplified seven-day physical-activity diary has provided valid estimates of physical activity in working women [30] and non-obese free-living adults [24], thus allowing the assessment of total daily energy expenditure and physical activity level. As stroke patients often demonstrate writing impairment and concentration difficulties, codes were used to indicate activities. The newly developed activity diary consisted of bundled sheets of paper, each containing a table with four columns:1) time, 2) activity 3) position and 4) intensity of the activity (TableS1). For each activity, patients were asked to record one number reflecting the main activity of the past 30 minutes. The main activity was defined as the activity that had taken the most time within the a 30-minute period. If two activities were performed for the same amount of time, participants were asked to report the most intense activity. The activity number was chosen from a list of 63 codes divided into six categories of activities: self-care, household tasks, work, therapy, leisure and home activities, and activities related to mobility and transport (TableS2). Additional numbers could be added for activities that were not included in the list. To avoid mistakes in recall, patients were instructed to complete the diary each time at the end of the 30-minute period. They were also instructed to record the position (lying, sitting or standing) in which each activity was performed. Finally the perceived intensity of each activity was rated along a rating scale of 6–20 [31]. Taking into account position and intensity, activities were converted in METs values, using the Compendium of Physical Activities Tracking Guide [32]. To calculate METs*minutes, Mets values were multiplied by 30 minutes. Mean METs values were subdivided into four levels, corresponding to sedentary (≤1 METs), light (>1 - <3 METs), moderate (3–6 METs) and vigorous activity (>6 METs) [33], [34]. In order to obtain **energy expenditure** in kcal/30 min, the following formula was used: [(METs-value×3.5×patient's weight)/200]×30minutes [35]. These results were multiplied by 24 to calculate energy expenditure over 12 hours (kcal/12 h).

According to the users manual the **SenseWear Pro2 Armband** (SWP2A) (HealthWear BodyMedia, Pittsburgh, PA, USA) should be worn on the right upper arm. For this study, however, it was worn on the non-hemiplegic upper arm positioned on the triceps muscle halfway between the acromion and the olecranon. The SWP2A was programmed using a computer interface, taking into account the participants' age, gender, height, weight, smoking habits and handedness prior to testing. This SWP2A contains two accelerometers, a galvanic skin response sensor, a heat flux sensor, a skin temperature sensor and a near-body ambient temperature sensor from which the data were stored minute by minute between 8:00 AM and 8:00 PM. Using a proprietary algorithm (Bodymedia, Sense Wear 6.1) the data were converted into **Metabolic Equivalents minutes** (METs*minutes) and **energy expenditure**. It has been validated for measuring energy expenditure in 50 healthy and diabetic participants against double-labeled water [36] and in 23 participants during light-intensity stepping in a Whole Room Calorimeter [37].

### Statistical Analysis

All data were analyzed using SPSS (version 20.0,SPSS Inc., Chicago). Descriptive statistics were calculated for patient characteristics. Normality was verified with the Kolmogorov-Smirnoff test. Because most of the data were not normally distributed, non-parametric statistics were used.

In order to study concurrent validity, a Spearman correlation coefficient (r_s_) was calculated to evaluate the relationship between the patient's diary and the observer's diary and between the patient's diary and the SWP2A. Values less than 0.30 were taken to indicate poor correlations, with values between 0.30 and 0.50 indicating low correlations, between 0.50 and 0.70 moderate correlations, between 0.70 and 0.90 high correlations and greater than 0.90 very high correlations [38]. Statistical significance was set at p<0.05.

To visualize the level of agreement between the patient's self-monitoring diary and both criterion standards (observer's diary; SWP2A) the values of the criterion standard were plotted against the difference between the two methods, thus providing an indication of agreement. The median and percentiles 25 and 75 were calculated.

## Results

### Descriptive Statistics

The research sample consisted of 16 patients with a mean age of 68 years (±11) and mean time since stroke of 78 days (±53). Four patients used no walking aids. Table 1 provides a description of the demographic and clinical characteristics of the patients. No data points were missing after recollection the diaries. When activities were missing, they were retrospectively added during the following day. Out of the 63 codes, the numbers which were frequently used were related to self-care (19.53%), therapy related activities (17.71%), resting in bed or in (wheel)chair (12.24%), watching television (9.38%), and talking (8.60%).

**Table 1:** Demographic and clinical characteristics of included patients.

Characteristics	Stroke = 16
Age at stroke onset, mean (y)±SD	68.31±10.95
Gender female, n (%)	7(43.8)
Height, mean (m)±SD	1.69±00.17
Weight, mean (kg)±SD	67.83±12.39
BMI, mean (kg/m^2^)±SD	25.42±05.08
Time since stroke, median (d)(IQR)	62.50(47.25)
Stroke type	
Ischemic, n (%)	9(56.3)
Hemorrhagic, n (%)	7(43.8)
Side of hemiparesis, right, n (%)	10(62.5)
Disability stroke	
RMA-GF, median (IQR)	7(5–11)
FAC, median (IQR)	3(2–5)
Mobility	
No use of walking aids in ADL, n (%)	4(25)

Abbreviations: d = days, SD =  standard deviation, % =  percentage, RMA-GF =  Rivermead Motor Assessment Gross Function, FAC =  Functional Ambulation Categories, n =  number, IQR =  Interquartile Range.

Almost every activity number was mentioned in the diaries except brushing hair, performing handicraft, and driving a car. A few new codes (N = 4) were listed, such as reading, smoking, resting in a (wheel)chair or in bed. Mostly activities were executed in sitting (84.1%) and standing position (9.9%). It concerned sedentary activities. Patients noted that help was required in 26.3% of all activities.

Table 2 provides summary of the results for METs*minutes and energy expenditure per 12 hours, as collected through the activity diaries of the patient and observer, as well as through activity monitoring. None of the patients performed vigorous activities.

**Table 2:** METs*minutes and Energy Expenditure values measured by two activity diaries and an Activity monitor in 16 stroke patients.

METs*minutes	Median	Minimum	Maximum	P25	P75
Diary patient					
Sedentary	342.00	120.00	420.00	247.50	378.00
Light	457.50	270.00	960.00	367.50	502.50
Moderate	397.50	300.00	1125.00	390.00	570.00
Vigorous	/	/	/	/	/
Total	1227.00	1134.00	1740.00	1184.25	1437.00
Diary researcher					
Sedentary	379.50	150.00	474.00	284.25	419.25
Light	405.00	225.00	765.00	300.00	603.75
Moderate	405.00	180.00	705.00	390.00	480.00
Vigorous	/	/	/	/	/
Total	1176.00	1080.00	1515.00	1123.50	1299.75
Activity monitor					
Sedentary	293.52	116.96	538.31	209.24	437.95
Light	521.82	5.88	768.17	345.30	575.41
Moderate	70.96	0.00	326.43	27.09	216.62
Vigorous	/	/	/	/	/
Total	896.16	486.74	1246.07	839.57	1026.45
**Energy Expenditure**	**Median**	**Minimum**	**Maximum**	**P25**	**P75**
Diary patient	1604.93	977.55	1927.80	1361.59	1610.44
Diary researcher	1473.78	967.26	1875.83	1249.63	1749.23
Activity monitor	965.33	728.12	1450.70	867.49	1056.56

Abbreviations: P25-P75 =  percentile 25–75. Metabolic Equivalents (METs)-values: Diary: METs- values per activity based on Compendium of Ainsworth^20^×30 minutes, subdivided in sedentary activity (≤1 METs), light activity (>1-<3 METs), moderate activity (3–6 METs), vigorous activity (>6 METs). Activity monitor: calculated by SenseWear Pro 2 armband. Energy expenditure kilocalories (kcal/12 h)-values: Diary: kcal/12 h calculated by ((METs valuereported per activity ×3.5×patients weight)/200×30minutes)^23^×24. Activity monitor: kcal/12 h calculated by SenseWear Pro 2 armband.

### Concurrent Validity

The correlation for METs*minutes in the diaries of the patients and the observers diaries was 0.75 (p<0.001), thus indicating a high correlation (Table 3). High correlations were also revealed for sedentary (r_s_ = 0.74,p<0.01) and moderate (r_s_ = 0.71,p<0.01) activity levels. A low and non-significant correlation was found for the activity category “light”. When the patients' activity diaries were compared to the SWP2A, the correlation coefficients were not significant.

**Table 3:** Spearman Rank Correlations between Patient's diary versus Researcher's Diary and versus Activity monitor in 16 stroke patients for physical activity (METs*minutes) and energy expenditure.

METs*minutes	Diary patient-Diary researcher	Diary patient-Activity monitor
Sedentary	0.74(p = 0.001)**	0.16 (p = 0.567)
Light	0.37 (p = 0.162)	0.11 (p = 0.691)
Moderate	0.71 (p = 0.002)**	0.22 (p = 0.410)
Vigorous	/	/
Total	0.75 (p = 0.001)**	0.15 (p = 0.590)

Energy expenditure	Diary patient-Diary researcher	Diary patient-Activity monitor
Total	0.92 (p = 0.000)**	0.29(p = 0.276)

Abbreviations: ** =  p<0.01.

**Figure 1:**
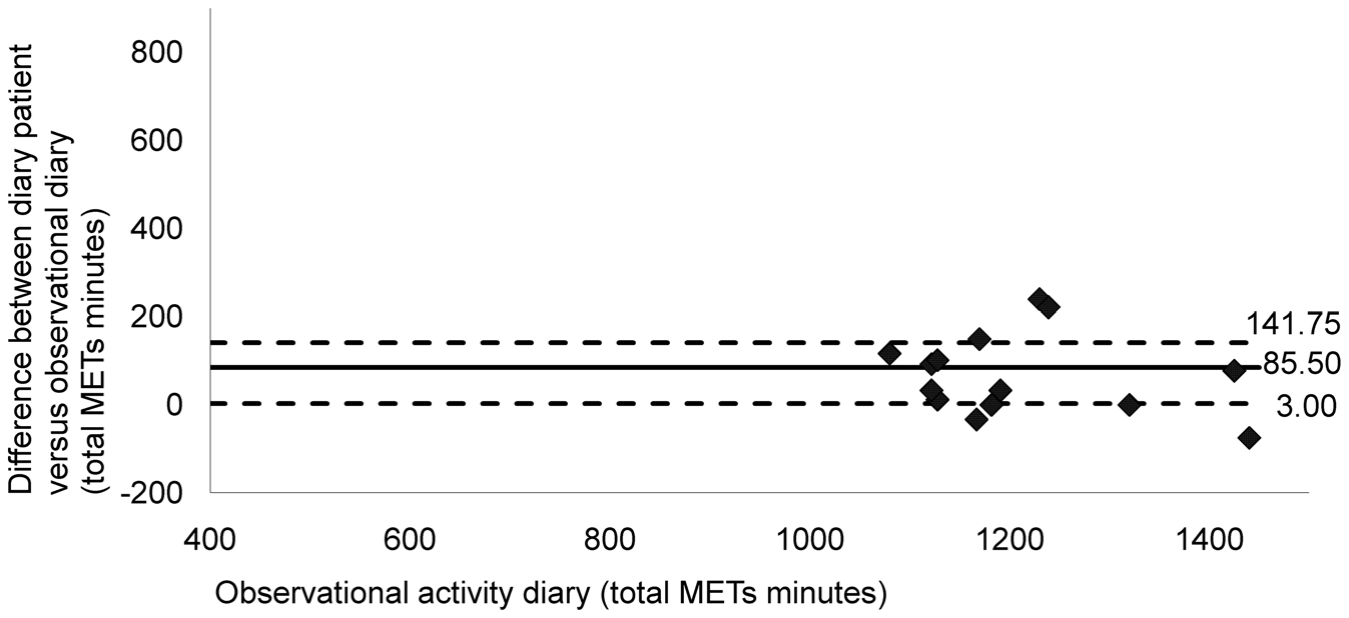
Comparing total Mets*minutes in 16 stroke patients: observational diary versus patient diary. Total Mets*minutes of observer activity diary was compared with diary of stroke patients. Broken horizontal lines represent percentiles 25 and 75, bold solid lines represent the median value of difference. Data analysis showed a good level of agreement between both diaries, data points clustering around zero (Median = 85.50; P25 = 3.00; P75 = 141.75). An underestimation of total METs*minutes for all patients is noted in comparison with the patient's diary. Visual inspection revealed no systematic bias.

**Figure 2:**
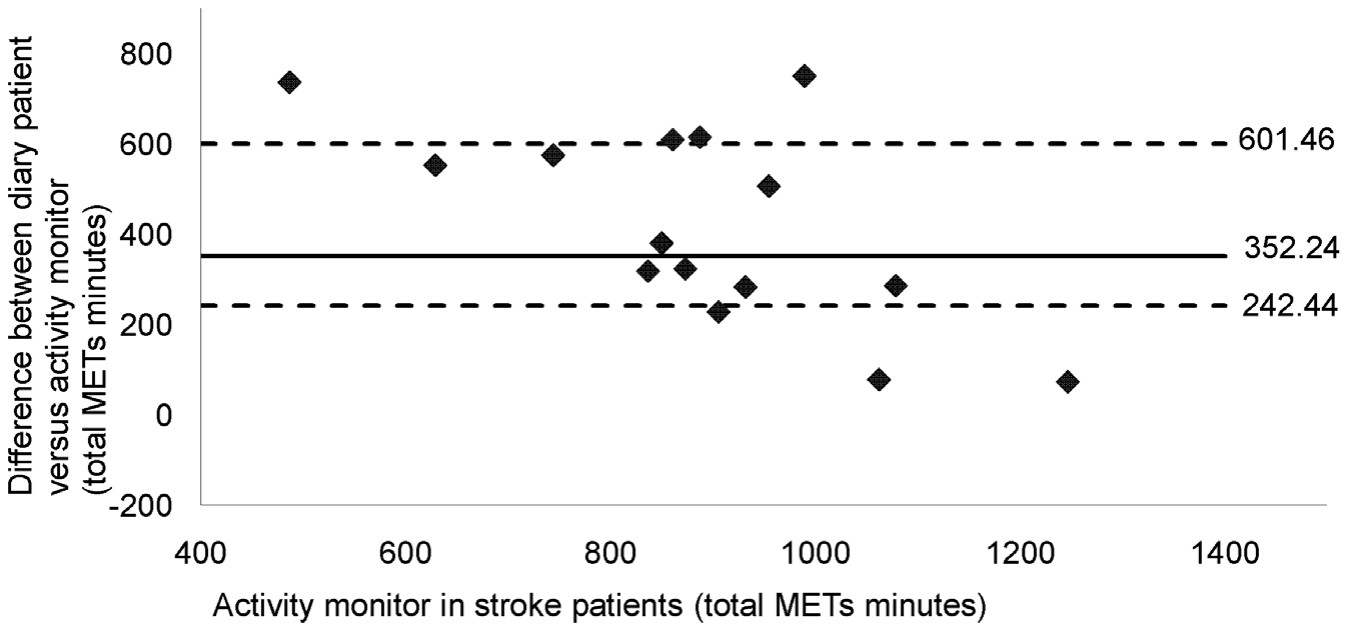
Comparing total Mets*minutes in 16 stroke patients: activity monitor versus patient diary. Total Mets*minutes of activity monitor was compared with diary of stroke patients. Broken horizontal lines represent percentiles 25 and 75, bold solid lines represent the median value of difference. Data analysis showed no good level of agreement between patient diary and the activity monitor (Median = 352.24; P25 = 242.44; P75 = 601.46). Visual inspection revealed no systematic bias.

Graphic analysis indicated a good level of agreement between both diaries (median value of the difference = 85.50; P25 = 3.00; P75 = 141.75) (Figure 1). Data points were clustered around zero. Less agreement was found between the patients' diaries and the SWP2A (median value of the difference = 352.24; P25 = 242.44; P75 = 601.46) (Figure 2). Lower total METs*minutes for all patients was observed in comparison with the patients' diaries. Visual inspection revealed no systematic bias.

Comparison of the data from the two diaries, revealed a very high correlation (r_s_ = 0.92, p<0.01) for energy expenditure, as measured between 8:00 AM and 8:00 PM (Table 3). Comparison between the patients' diaries and the SWP2A revealed a poor correlation (r_s_ = 0.29, p<0.01) with regard to energy expenditure.

**Figure 3:**
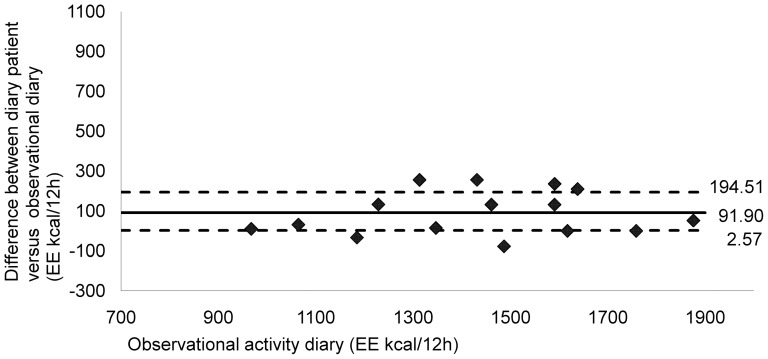
Comparing energy expenditure in 16 stroke patients: observational diary versus patient diary. Energy expenditure (kcal/12 h) of observer activity diary was compared with diary of stroke patients. Broken horizontal lines represent percentiles 25 and 75 value, bold solid lines represent the median value of difference. Data analysis showed good agreement between both diaries (Median = 91.90; P25 = 2.57; P75 = 194.51). Most data are clustered around the zero point.

**Figure 4:**
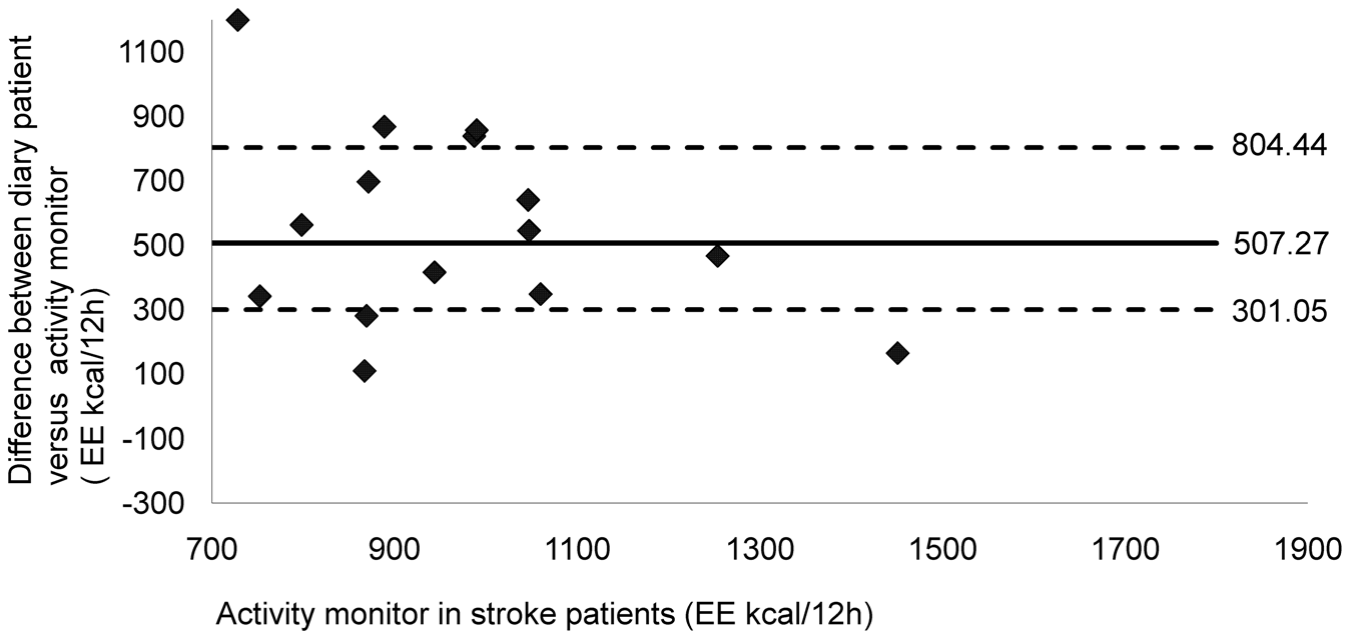
Comparing energy expenditure in 16 stroke patients: activity monitor versus patient diary. Energy expenditure (kcal/12 h) of observer activity diary was compared with diary of stroke patients. Broken horizontal lines represent percentiles 25 and 75 value, bold solid lines represent the median value of difference. The activity monitor is underestimating data for all patients in comparison to the diary filled in by the patient (Median = 507.27; P25 = 301.05; P75 = 804.44).

Graphic analysis of the data concerning total energy expenditure indicated good agreement between the two diaries (median value of the difference = 91.90; P25 = 2.57; P75 = 194.51) (Figure 3). Most of the data were clustered around the zero point. The SWP2A underestimated energy expenditure for all patients, in comparison to the diaries completed by the patient (median value of the difference = 507.27; P25 = 301.05; P75 = 804.44) (Figure 4).

## Discussion

This study assessed the concurrent validity of a coded self-monitoring activity diary for measuring activity level and total energy expenditure in hospitalized stroke patients. The diary generated valid results in comparison to the diary kept simultaneously by an observer, as used to determine sedentary physical activity (<1METs), moderate physical activity (3-6 METs) and total physical activity over 12 daytime hours. A very high correlation between the two diaries was observed for total energy expenditure during daytime hours. Poor correlations were observed, however, when comparing the diary to the SWP2A for measuring activity level and energy expenditure.

A high correlation was found between the two diaries, when measuring *sedentary and moderate* physical activities during daytime hours, while a low correlation was found for light activities. One possible explanation is that activities in the levels of sedentary and moderate activities are more easily recalled than are light activities. Sedentary activities include activities in very low intensity (e.g., sleeping or sitting quietly), which are often longer in duration. Moderate activities are more intense (e.g., such as physical therapy or occupational therapy), and they are well reported in the daily schedules of rehabilitation centers. The lack of a good correlation between the two diaries with regard to *light* physical activities could be that these relatively brief activities (e.g., talking, grooming, reading) of limited duration which are often less planned and remembered than are activities of other levels. Another explanation might have to do with outliers. The scores entered by two patients differed excessively from those entered by the other patients and by the observers. When the Spearman correlation was recalculated excluding the data from these two patients, a moderate correlation for light activities was observed (r_s_ = 0.63, p<0.05). The correlations for sedentary and moderate levels, however, remained slightly higher. Moderate activity is considered an important activity level in stroke rehabilitation, as it may be sufficient to produce a significant reduction in stroke risk [2], [39]. With regard to total energy expenditures, comparison of the two diaries revealed a very high correlation. These results thus indicate that self-reported coded-activity diaries constitute a potentially valuable method for use in clinical settings, but that they should be explored further in relation to an objective “gold standard”.

Comparisons of the patients' diaries to the SWP2A data revealed poor correlations. In general, the activity monitor reported lower values for 12-hour energy expenditure than did either diary. One explanation might be that the activity diary over-estimated the energy expenditure and the time in moderate activity as time intervals were long and only one activity was allowed to be reported every 30 minutes. This would suggest a need to shorten the time intervals in which activities are reported. The SWP2A has not been validated to measure energy expenditure against a “gold standard” method in stroke patients, who have inefficient gait patterns causing higher cost of energy for given activity. As such the SWP2A may have underestimated total energy expenditure and time in moderate physical activity. It would be advisable to develop patient-specific algorithms to accurately use the SWP2A in stroke patients.

This study is the first study to use a coded self-monitoring activity diary to assess physical activity in stroke patients. Because understanding the instructions of the diary requires a certain level of comprehension, patients with severe cognitive deficits were excluded. No inconvenience was reported. This study showed no missing data points, because all missing activities were collected by an interviewer through recollection, and the previously completed periods facilitated this. We estimate that less than 10% of the activities were missing in the diaries. Missing data often concerned periods during which patients performed sedentary and light physical activities (e.g., reading, resting, watching television) of long duration (<3 METs). Also evening activities were sometimes forgotten, which could be attributed to the fact that nursing care started at this time moment. In many cases, only one 30-minute period was completed. Patients started filling in the diaries when a new activity started. Considering the fact that the missing activities were filled in at the next day and the type of missing activities were easy to remember and low in percentage, we think that this diary is well applicable in stroke patients.

Completing the diary can be a tool for helping patients and family/caregivers reflect on the types of activity level, possibly leading to increases in the activity level. When evaluating the clinical relevance of the results of this study, it is important to note the lack of severe stroke patients in the research sample. Also in this study, no activities were categorized as vigorous by any of the three measurement tools. This is not surprising as aerobic exercises are seldom integrated as part of neuro-motor rehabilitation programs despite evidence supporting the importance [3]. Previous research strongly suggests that aerobic training is necessary in stroke rehabilitation, however, and that it should be supplemented with strength-developing exercises for both lower limbs [40]–[42].

Further research is required before the self-monitoring coded-activity diary can be implemented into clinical practice. Studies with objective criterion standards, (e.g., such as doubly labeled water or indirect calorimetry) and the development and examination of a digitized version are recommended. It might also be advisable to delete the fourth column of the diary, where the perceived intensity of an activity was marked on a rating scale of 6–20. This seemed difficult for patients to fill in. Also this could not be retrospectively added. Also it might be recommendable to shorten the time intervals in which activities are reported, to collect data for periods longer than one day, to ask patients about inconvenience filling in the diary, and provide an additional day to familiarize patients with the process of completing a diary.

## Conclusions

Coded self-monitoring activity diaries appear feasible as a low-tech alternative to labor-intensive observational diaries for determining sedentary, moderate, and total physical activity and for quantifying energy expenditure in hospitalized stroke patients. Given the poor correlation with the objective measurement of physical activity, however, further research is needed to validate its use against a gold standard measure of physical activity intensity and energy expenditure (e.g., doubly labeled water or indirect calorimetry).

## Supporting Information

Table S1 Activity diary.
(DOCX)

Table S2 Scheme of codes that are used to fill in the activity diary.
(DOCX)
