# Supplementary material for: Is a Coded Physical Activity Diary Valid for Assessing Physical Activity Level and Energy Expenditure in Stroke Patients?
Source: PLoS One. 2014 Jun 6;9(6):e98735. doi: 10.1371/journal.pone.0098735 (PMC4048313; doi:10.1371/journal.pone.0098735)
Supplement: Table S1 — Activity diary. (DOCX) [file pone.0098735.s001.docx]

Table S1: Activity diary

| **Duration** | | **Activity number** | **Position** | **How intense was the activity?** |
| --- | --- | --- | --- | --- |
| 8h00 | 8h30 |  | Lying, Sitting, Standing | 6-7-8-9-10-11-12-13-14-15-16-17-18-19-20 |
| 8h30 | 9h00 |  | Lying, Sitting, Standing | 6-7-8-9-10-11-12-13-14-15-16-17-18-19-20 |
| 9h00 | 9h30 |  | Lying, Sitting, Standing | 6-7-8-9-10-11-12-13-14-15-16-17-18-19-20 |
| 9h30 | 10h00 |  | Lying, Sitting, Standing | 6-7-8-9-10-11-12-13-14-15-16-17-18-19-20 |
| 10h00 | 10h30 |  | Lying, Sitting, Standing | 6-7-8-9-10-11-12-13-14-15-16-17-18-19-20 |
| 10h30 | 11h00 |  | Lying, Sitting, Standing | 6-7-8-9-10-11-12-13-14-15-16-17-18-19-20 |
| 11h00 | 11h30 |  | Lying, Sitting, Standing | 6-7-8-9-10-11-12-13-14-15-16-17-18-19-20 |
| 11h30 | 12h00 |  | Lying, Sitting, Standing | 6-7-8-9-10-11-12-13-14-15-16-17-18-19-20 |
| 12h00 | 12h30 |  | Lying, Sitting, Standing | 6-7-8-9-10-11-12-13-14-15-16-17-18-19-20 |
| 12h30 | 13h00 |  | Lying, Sitting, Standing | 6-7-8-9-10-11-12-13-14-15-16-17-18-19-20 |
| 13h00 | 13h30 |  | Lying, Sitting, Standing | 6-7-8-9-10-11-12-13-14-15-16-17-18-19-20 |
| 13h30 | 14h00 |  | Lying, Sitting, Standing | 6-7-8-9-10-11-12-13-14-15-16-17-18-19-20 |
| 14h00 | 14h30 |  | Lying, Sitting, Standing | 6-7-8-9-10-11-12-13-14-15-16-17-18-19-20 |
| 14h30 | 15h00 |  | Lying, Sitting, Standing | 6-7-8-9-10-11-12-13-14-15-16-17-18-19-20 |
| 15h00 | 15h30 |  | Lying, Sitting, Standing | 6-7-8-9-10-11-12-13-14-15-16-17-18-19-20 |
| 15h30 | 16h00 |  | Lying, Sitting, Standing | 6-7-8-9-10-11-12-13-14-15-16-17-18-19-20 |
| 16h00 | 16h30 |  | Lying, Sitting, Standing | 6-7-8-9-10-11-12-13-14-15-16-17-18-19-20 |
| 16h30 | 17h00 |  | Lying, Sitting, Standing | 6-7-8-9-10-11-12-13-14-15-16-17-18-19-20 |
| 17h00 | 17h30 |  | Lying, Sitting, Standing | 6-7-8-9-10-11-12-13-14-15-16-17-18-19-20 |
| 17h30 | 18h00 |  | Lying, Sitting, Standing | 6-7-8-9-10-11-12-13-14-15-16-17-18-19-20 |
| 18h00 | 18h30 |  | Lying, Sitting, Standing | 6-7-8-9-10-11-12-13-14-15-16-17-18-19-20 |
| 18h30 | 19h00 |  | Lying, Sitting, Standing | 6-7-8-9-10-11-12-13-14-15-16-17-18-19-20 |
| 19h00 | 19h30 |  | Lying, Sitting, Standing | 6-7-8-9-10-11-12-13-14-15-16-17-18-19-20 |
| 19h30 | 20h00 |  | Lying, Sitting, Standing | 6-7-8-9-10-11-12-13-14-15-16-17-18-19-20 |
