# Supplementary material for: Is a Coded Physical Activity Diary Valid for Assessing Physical Activity Level and Energy Expenditure in Stroke Patients?
Source: PLoS One. 2014 Jun 6;9(6):e98735. doi: 10.1371/journal.pone.0098735 (PMC4048313; doi:10.1371/journal.pone.0098735)
Supplement: Table S2 — Scheme of codes that are used to fill in the activity diary. (DOCX) [file pone.0098735.s002.docx]

Table S2: Scheme of codes that are used to fill in the activity diary

| **Codes** | **Activities** |
| --- | --- |
| **Self-care** | |
| 1 | Dressing/ undressing |
| 2 | Physical care (eg brushing teeth, shaving, washing at a sink, make-up, combing hair) |
| 3 | Bathing |
| 4 | Shower |
| 5 | Brushing hair |
| 6 | Toilet use |
| 7 | Eating |
| **Household activities** | |
| 8 | Making bed |
| 9 | Vacuuming |
| 10 | Cleaning |
| 11 | Food preparations, cooking |
| 12 | Covering table, clear table |
| 13 | Washing dishes or drying |
| 14 | Shopping |
| 15 | Putting away groceries |
| 16 | Doing laundry, folding or hanging clothes, putting clothes in washer or dryer, putting away clothes |
| 17 | Ironing |
| 18 | Watering plants |
| 19 | Getting wood |
| 20 | Walking in house |
| 21 | Playing with child(ren) |
| 22 | Playing with animals |
| 23 | Child care: dressing/bathing/ grooming/ feeding/ occasional lifting |
| **Work and related activities** | |
| 24 | Occupation |
| 25 | Volunteering |
| 26 | Regulating financial affairs |
| 27 | Repairing |
| 28 | Following a course |
| **Therapy** | |
| 29 | Physiotherapy in group |
| 30 | Individual physiotherapy |
| 31 | Occupational therapy in group |
| 32 | Individual occupational therapy |
| 33 | Speech therapy |
| 34 | Psychology |
| 35 | Nursing care |
| 36 | Doctor care |
| 37 | Other:…………………………………………………………………………………………………………………………. |
| **Leisure activities and home activities** | |
| 38 | Playing music |
| 39 | Surfing on computer, Financial affairs on computer |
| 40 | Talking to persons directly or by phone |
| 41 | Playing board games, cards |
| 42 | Doing puzzles |
| 43 | Drawing |
| 44 | Writing |
| 45 | Performing handicraft (knitting,.........) |
| 46 | Watching television, listening to music |
| 47 | Sitting |
| 48 | Cinema and theater going, other trips |
| 49 | Sleeping |
| 50 | Mowing lawn |
| 51 | Weeding |
| 52 | Shoveling |
| 53 | Bicycling |
| 54 | Swimming |
| 55 | Conditioning exercises |
| 56 | Walking outside |
| 57 | Sexual activities |
| **Mobility, transport related activities** | |
| 58 | Driving a car |
| 59 | Travelling with someone / public transport |
| 60 | Walking short distances |
| 61 | Driving a wheelchair |
| 62 | Pushed in wheelchair |
| 63 | Climbing stairs |
| Here additional numbers could be added, when an activity was not found in the list | |
| 64 |  |
| 65 |  |
| 66 |  |
| 67 |  |
| 68 |  |
| 69 |  |
| 70 |  |
| 71 |  |
| 72 |  |
| 73 |  |
| 74 |  |
| 75 |  |
| 76 |  |
| 77 |  |
| 78 |  |
| 79 |  |
| 80 |  |
